# Setting Time, Microstructure, and Durability Properties of Low Calcium Fly Ash/Slag Geopolymer: A Review

**DOI:** 10.3390/ma15030876

**Published:** 2022-01-24

**Authors:** Salem Aldawsari, Raphael Kampmann, Jörg Harnisch, Catharina Rohde

**Affiliations:** 1 Department of Civil & Environmental Engineering, FAMU-FSU College of Engineering, Tallahassee, FL 32310, USA; 2 Department of Civil Engineering, Prince Sattam Bin Abdulaziz University, Al-Kharj 11942, Saudi Arabia; 3 Materialprüfanstalt Schleswig-Holstein, Technische Hochschule Lübeck, 23562 Lübeck, Germany; raphael.kampmann@th-luebeck.de; 4 Department of Civil Engineering, University of Applied Sciences, 48149 Münster, Germany; j.harnisch@fh-muenster.de; 5 Department of Civil Engineering, Bauhaus-Universität Weimar, 99423 Weimar, Germany; catharina.rohde@uni-weimar.de

**Keywords:** geopolymer, alkali activated, review, fly ash, slag, permeability, shrinkage, chloride, sulfate, carbonatation

## Abstract

Ordinary Portland cement (OPC) is known for its significant contribution to carbon dioxide emissions. Geopolymer has a lower footprint in terms of CO_2_ emissions and has been considered as an alternative for OPC. A well-developed understanding of the use of fly-ash-based and slag-based geopolymers as separate systems has been reached in the literature, specifically regarding their mechanical properties. However, the microstructural and durability of the combined system after slag addition introduces more interactive gels and complex microstructural formations. The microstructural changes of complex blended systems contribute to significant advances in the durability of fly ash/slag geopolymers. In the present review, the setting time, microstructural properties (gel phase development, permeability properties, shrinkage behavior), and durability (chloride resistance, sulfate attack, and carbonatation), as discussed literature, are studied and summarized to simplify and draw conclusions.

## 1. Introduction

Building materials have evolved over centuries during the history of construction to improve various housing needs and to meet contemporary demands. One of these evolving materials is concrete, which has survived for centuries due to its high performance and long lifespan. Concrete is a composite material, primarily made from ordinary Portland cement, water, coarse aggregate, and fine aggregate. Nowadays, Portland cement is the main binder for ordinary concrete (OPC) and it has been produced extensively as it is considered one of the most dominant binders in the building industry. In the nineteenth century, when producers and consumers demanded strength and stabilization despite their pronounced environmental effects, Portland cement was the breakthrough technology and contributed to advances in the field of materials science [1]. The excessive production of Portland cement contributes to high amounts of carbon dioxide (CO_2_) being released into the atmosphere. Carbon dioxide is a notable pollutant and contributes approximately 0.95 tons of CO_2_ emissions for each ton of Portland cement produced [2]. The Portland cement industry contributes significantly to CO_2_ emissions globally, with values ranging from 5% to 7% [3]. Although the demand for Portland cement continues to exist, concern about environmental issues is rising, and the affected industries are currently identifying alternative solutions in the light of global warming [4].

Throughout the past decades, researchers have shifted more toward sustainable materials through either the partial or full replacement of OPC with more environmentally friendly alternatives. Some of the most promising alternatives, which may replace significant volumes of Portland cement in the future, are geopolymer materials due to their low shrinkage, sulfate resistance, early strength gain, corrosion resistance, and long-term properties [5]. In the 1970s, Joseph Davidovits [6] developed and named geopolymer; however, the constituents of similar materials have evolved since the 1950s in the Soviet Union, where the term “soil cement” was used [7]. Geopolymer materials consist primarily of an aluminosilicate source and liquid alkaline constituents. Alkaline liquids are used to activate the aluminosilicate materials to form three-dimensional structure products. Aluminum and silicate exist abundantly in nature or can easily be obtained from industrial by-products such as fly ash, blast furnace slag, rice husk ash, and other materials that offer sufficient alumina and silica content to provide pozzolanic properties. Alkaline solutions such as sodium hydroxide (NaOH), sodium silicate (Na_2_SiO_3_), potassium hydroxide (KOH), and potassium silicate (K_2_SiO_3_) are commonly used activators. Alkaline activators are used to liberate silicon (Si) and aluminium (Al) from the aluminosilicate species. When Si and Al are released, a supersaturated aluminosilicate solution is created, which subsequently contributes to gel formation with large complex networks [8]. Davidovits [9] defined the three-dimensional structure of silico-aluminate by semi-crystalline to amorphous of types poly(sialate) (-Si-O-Al-O-), poly(sialate-siloxo) (-Si-O-Al-O-Si-O-), poly(sialate-disiloxo) (-Si-O-Al-O-Si-O-Si-O-), and he suggested a general empirical formula of polysialate:

(1) $$M_{n}{\left[-{\left({\mathrm{SiO}}_{2}\right)}_{z}-{\mathrm{AlO}}_{2}\right]}_{n},wH_{2}O$$

where M is the alkaline element, *n* is the degree of polymerization, and *z* takes on one of the following values—1, 2, or 3. Geopolymer materials have been found to possess similar or superior properties (strength, shrinkage, fire resistance, and chemical resistance) when compared to ordinary Portland cement [10,11,12,13,14]. Though geopolymer materials have been heavily researched throughout the recent decades and new developments have led to significant improvements, the lack of standards and long-term experience have increased the uncertainty about the ability of geopolymers to survive for a long-term lifespan.

Although OPC has been researched and successfully used for almost 170 years, the molecular-scale mechanism that occurs during OPC hardening is still not fully understood [15]. Portland cement had been studied and researched for decades until it advanced to what is seen today in industrial, residential, and commercial buildings, and yet it has an established reputation and researchers have obtained a well-developed understanding of its characteristics. As geopolymer research has taken many different directions and paths over the years, it is crucial to reevaluate the state of the art and comprehensively summarize the most significant findings.

**Figure 1:**
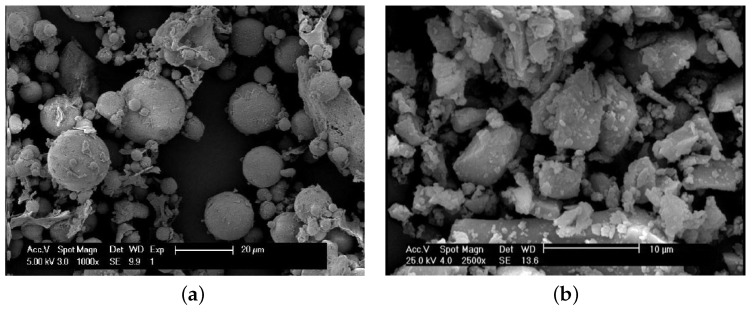
Particle shapes of (**a**) fly ash (Reprinted with permission from Ref. [18]. Copyright 2012 Ann E. Benbow) and (**b**) ground-granulated blast-furnace slag (GGBFS) (Reprinted with permission from Ref. [19]. Copyright 2018 Elsevier).

Fly ash and slag have been used extensively as raw materials in geopolymers, either in combination with each other or separately, and are the focus of this review. Because the presented work reflects on fly ash and slag as the primary binders, a brief introduction to these two materials in regard to their production, as well as their physical and compositional properties, is presented below. Fly ash is a by-product formed during the burning process of pulverized coal for the generation of electricity and has pozzolanic properties that make it suitable for use in concrete. Fly ash is defined by ASTM-International [16] as “finely divided residue that results from the process of combustion of ground or powdered coal and that is transported by flue gases”. Because fly ash particles solidify in the air, where they seek the lowest energy state, they are spherical in nature, as shown in Figure 1a, and fine spherical particles are separated from flue gases by collective systems or electrostatic participators [17].

Fly ash mainly consists of silicon (SiO_2_), aluminum (Al_2_O_3_), iron (Fe_2_O_3_), and calcium oxide (CaO), as well as some minor components such as potassium, magnesium, sodium, sulfur, and titanium. The most common fly ashes are classified by ASTM-International [20] as class F fly ash and class C fly ash. Class F fly ash has a low calcium oxide (CaO) content, which differentiates it from class C fly ash, with a higher amount of CaO. The CaO content for class F fly ash should not exceed a maximum of 18%, whereas class C may exceed this value, according to [20]. Both classes must contained a minimum of 50% silica, aluminum, and iron contents, and a maximum of 5%, 3%, and 6% for sulfur trioxide (SO_3_), moisture content, and loss on ignition, respectively. These values and limits do not predict the performance of the fly ash, but rather help in characterizing the uniformity and the composition of the material [20].

Steel slag is formed during the manufacturing of iron and steel as a by-product that mainly consists of SiO_2_, Al_2_O_3_, Fe_2_O_3_, CaO, magnesium oxide (MgO), and minor amounts of sulfur trioxide (SO_3_), titanium dioxide (TO_2_), and potassium oxide (K_2_O). The formation of steel slag occurs during iron manufacturing in a basic oxygen furnace (BOF) or during steel production from scrap in an electric arc furnace (EAF). In a BOF, oxygen is used to expel impure components such as silicon, carbon, phosphorus, and manganese. These impurities then join with lime and dolomitic lime to create slag. In EAF, liquid slags floats on top of the molten steel, which can be separated and dispersed by tilting the EAF such that the upper layer of slag leaves the EAF, whereas the molten steel remains in the vessel. The molten material is skimmed off and left to cool down to become slag [21]. For granulated blast-furnace slag with cementitious properties, the manufacturing depends on the rapid quenching of the molten slag by means of water or air quenching to ensure that the material reaches the glassy state. Water quenching using high-pressure water jets is considered the most effective process because it produces a higher amount of glass structures in the slag [22]. After quenching the molten slag by means of powerful water jets in the granulator, granulated blast furnace slag (GBFS) is formed [22], and then due to the grinding process, ground granulated blast furnace slag (GGBFS) particles are mostly angular, as shown in Figure 1b. ASTM-International [16] defines this material as “the glassy, granular material formed when molten blast-furnace slag is rapidly chilled, as by immersion in water”.

In addition to the processing conditions, variations in the constituent materials of geopolymers, such as fly ash, slag, and alkaline liquids, contribute significantly to the different physical properties and characteristics of geopolymers. For example, shrinkage, fire resistance, setting time, thermal conductivity, and compressive strength [8] are all affected by the properties of the individual constituent materials. The addition of ground granulated blast furnace slag to fly ash-based geopolymers has enhanced their mechanical properties [13,23], reduced setting time [24,25,26] and leading to decreased permeability [23,27,28]. However, replacing fly ash partially with GGBFS produces more gel phases and complex system structures, with increased shrinkage [29,30]. Furthermore, GGBFS, alkaline activator, and the activator concentration substantially affect the setting time. The correlation between these factors and setting time requires further evaluation to achieve a comprehensive overview of the preceding research studies. In this context, it seems essential to find the optimum replacement of fly ash by slag [24,26,29,31], alkaline liquid ratios [24,31,32], solution concentrations [26,31], and key parameters that influence the blended system to attain an optimum setting time and to better predict how these systems form and behave in engineering applications. Accordingly, this paper targets the use of low-calcium class-F fly ash in combination with ground granulated blast furnace slag because the separate use of fly ash-based or slag-based geopolymers are well-developed topics in the literature, whereas the heterodyne effect has not yet been properly studied. Although the mechanical properties of the blended fly ash and slag system has been researched extensively [33,34,35,36,37], its microstructure and durability are not fully established topics and require further evaluation.

In the present article, we revisited seventy-nine research studies, and approximately sixty of these studies were published between 2010 and 2020. The studied literature is focused on binary systems with low-calcium fly ash (Class F) and slag as the primary binders. In this study, the influential parameters that affect the initial and final setting time were evaluated and tabulated. In addition, in this study we also focused on the significant findings concerning microstructural properties (gel phase development, permeability properties, shrinkage behavior) and durability properties (chloride resistance, sulfate attack, and carbonatation) that were extensively studied and discussed.

## 2. Research Significance

Though fly ash-based and slag-based geopolymers have been extensively evaluated in their separate forms, the engineering properties, microstructure developments, and durability studies for combined systems require further evaluations due to the complexity of the formed structures. Despite the fact that the combination of fly ash and slag as a geopolymer system improved permeability [13,23,27] and mechanical properties [27,34,36] and led to a more controlled expedition of setting time [24,25,26], combined systems produce more complex structures and gel phases that require further evaluation to attain a good understanding of these materials’ short- and long-term lifespans.

The previous published studies available in the literature have mainly focused on the mechanical properties of geopolymers with low-calcium class-F fly ash or ground granulated blast furnace slag (GGBFS) as separate systems, with few studies focused on combined systems. However, the microstructure and durability of blended systems have not yet been holistically analyzed, and this review will provide a detailed synopsis of what has been established in this type of geopolymers. Accordingly, this review paper evaluates what has been reported and achieved in the accessible literature, with a focus on setting time, microstructure, and durability to centralize the available information and to draw state-of-the-art conclusions. The presented study seeks to provide important comprehensive and contextual information about the changes in the characteristics of blended geopolymer systems to address the complexity of the hydrated products, as well as their permeability, shrinkage behavior, chloride resistance, sulfate attack, and carbonatation properties.

## 3. Research Methodology

The setting time, microstructure, and durability are all essential categories in regard to concrete because they control the workability of fresh concrete and the durability of hardened concrete. When combining slag and fly ash to create a composite system, fast and slow chemical reactions occur and develop reaction products that contribute to significant changes in setting time [38], microstructure development [26,39], and the durability of the combined system [10,40]. The setting time of low-calcium fly ash is considered slow and needs to be accelerated to achieve a considerable strength at an early age [32]. Acceleration can be achieved by using heat [25,41] or through the addition of slag [25,29]. Using heat requires energy, whereas the addition of slag accelerates the setting at an ambient temperature and the energy used for heat is conserved. Therefore, knowledge about the factors affecting the setting time of fly ash/slag geopolymer systems is essential in order to control and optimize the fresh concrete. Moreover, the constituent materials of the activator solution and the addition of slag contribute to significant changes in the microstructure, which ultimately affect the durability of this system. Sodium and potassium hydroxides (NaOH and KOH), as well as sodium and potassium silicates (Na_2_SiO_3_ and K_2_SiO_3_) are commonly used activators for fly-ash-based geopolymers, slag-based geopolymers, or blended systems. NaOH to Na_2_SiO_3_ or KOH to K_2_SiO_3_ solution ratios, the fly ash/slag ratio, the liquid/binder ratio, and other additives such as superplasticizers contribute to the overall phase formation and performance of the blended system. The reaction products within the microstructure become more complex through the addition of slag, which positively influences the material characteristics such as shrinkage, permeability, chloride resistance, sulfate attack, and carbonatation. Optimizing these parameters is important in attaining a well performing blended system.

Although the published literature on fly-ash-based and slag-based geopolymers—in their separate forms—appears to be well developed, a full description of the material characteristics and the performance of combined systems deserves further evaluation. Because the addition of slag to a fly ash-based geopolymer introduces more intricate reaction products and adds a layer of complexity to the microstructural system, this study consolidates and evaluates the literature for combined fly ash-slag systems, including different activator types or dosages, additives used, different proportions, and other factors such as superplasticizers. It should be noted that the focus of this paper is on the use of low-calcium class-F fly ash and ground granulated blast furnace slag (GGBFS) as raw materials, as well as sodium hydroxide and sodium silicate as alkaline activators, because these materials represent the most published studies in the literature. In the following, these materials are analyzed separately to assess their effect on the whole system, with a focus on setting time, microstructural characteristics, and durability properties.

## 4. Setting and Hardened Characteristics

### 4.1. Setting Time

Setting time is an essential parameter that controls the duration of the workability with which the concrete can be placed and compacted into its form. The setting time of concrete should be sufficient enough for concrete to be handled and casted before any sign of setting. The low-calcium class-F fly ash-based geopolymer has a high setting time [32], which can be reduced significantly by using heat curing [25,41] or by adding GGBFS [25,29]. Evaluating the parameters that influence the setting time of these types of geopolymer is essential to control the setting duration and reach a setting that meets the desired time. The setting time of the combined system is mostly controlled by the FA/GGBFS ratio [25,29,35], the sodium silicate-to-sodium hydroxide (SS/SH) ratio [24,32], and the concentration of NaOH [26,31]. GGBFS has a short setting time and is used to expedite the setting time of fly ash. The effect of the SS/SH ratio corresponds to the SiO_2_ content provided by the alkaline solution, since the immediate availability of the soluble silica expedites the polymerization of the raw materials [42]. Furthermore, the molarity of NaOH contributes considerably to the setting time of fly-ash-based geopolymer. The higher the concentration of NaOH, the lower the setting time. The acceleration of the setting time resulting from a higher concentration of NaOH is attributed to the extra content of hydroxide ions OH^−^, which expedite the dissolution of fly ash at early stages of the reaction [43].

Nath and Sarker [32] studied GGBFS/fly ash geopolymer, in which fly ash was replaced by 10%, 20%, and 30% of slag. Three different ratios of sodium silicate-to-sodium hydroxide (SS/SH) of 1.5, 2.0, and 2.5 were used, with ratios of the alkaline solution to the binder of 0.35, 0.40, and 0.45, whereas the molarity of NaOH was kept constant at 14 M. The study concluded that using 10%, 20%, and 30% of slag content decreased the setting time from more than 24 h (100% fly ash) to 290 min, 94 min, and 41 min, respectively. In addition, the alkaline solution-to-binder ratio led to a considerable increase in setting time (approximately 33%) for each increment of the alkaline solution. Decreasing the ratio of SS/SH from 2.5 to 1.5 revealed an increase in the setting time. Jang et al. [25] conducted a study that included polycarboxylate-based and naphthalene-based superplasticizers and ground granulated blast furnace slag as factors to evaluate the compositional effects on the setting times. By adding 4% of polycarboxylate-based superplasticizer, the initial and final setting time was retarded by 50 and 70 min, respectively, whereas the incorporation of naphthalene-based superplasticizer from 1% to 4% did not reveal an effect on the setting time. Setting time was notably affected by the addition of GGBFS from 0% to 30%; in those cases, the change in setting time decreased more steadily from 30% to 100% of GGBFS content.

Hadi et al. [24] evaluated the effect of the addition of GGBFS, different SS/SH ratios, the alkaline-solution-to-binder ratios, and free water on the properties of fly ash geopolymer. It was found that with increasing slag content in the mix, the initial and final setting times decreased. In addition, the initial and final setting times decreased with an increasing SS/SH ratio. This was due to the increased soluble silica in the alkaline solution, which affected the crystallization and polymerization to produce a final gel with more stable and ordered structures due to the formation of a higher percentage of Si−O bridge bonds [42]. Kumar et al. [35] studied the influence of GGBFS on the reaction and final hydrated products of fly ash geopolymers. The alkaline activator used was sodium hydroxide with constant molarity at 6 M, and the alkaline-solution-to-binder ratio was 0.35. Fly ash was replaced with 0%, 5%, 10%, 15%, 20%, 25%, 35%, and 50% of GGBFS. The setting time using 100% of fly ash was recorded at 295 min, which was significantly reduced to 105 min by adding 5% of GGBFS. It was found that the setting time decreased gradually from 105 min to a minimum value of 45 min for 25%, 35%, and 50% of GGBFS replacement. The notable drop in the setting of fly ash using minimal or maximal amounts of GGBFS could be attributed to a rapid dissolution of GGBFS, and then the precipitation of C-S-H gel due to the activation of GGBFS.

Generally, setting time is significantly influenced by the slag content of the mixture; with increasing slag content, the setting time decreases. Alkaline liquid affects the setting time due to the silica that is soluble in the activator, in which the setting time is altered, showing a gradual drop when the SS/SH ratio increases. Other parameters that retard or expedite fly ash/slag geopolymer setting times include molarity, the solution/binder ratio, and superplasticizers. Based on the information available in the current literature, Table 1 summarizes the effect of GGBFS content, SS/SH ratio, the sodium hydroxide concentration, and other influential parameters.

### 4.2. Microstructural Properties

Fly ash-based and slag-based geopolymers have common characteristics, such as resilience in high-temperature environments and high compressive strength [1,44]. Although fly ash-based geopolymers offer several advantageous characteristics, such as resistance to acidic attacks and slow reactivity at room temperature [25,44], slag-based geopolymers provide benefits such as short setting time and reduced porosity, which is less than the porosity of comparable Portland cement mixtures [1]. Slag-based geopolymers reach higher mechanical strengths with lower initial permeability. As the material ages and matures, permeability increases due to the propagation of micro-cracks, whereas low calcium fly ash-based geopolymer becomes less permeable over time—similarly to OPC concrete [45]. The microstructure of the combined system is more complex due to the interacting multi-gel phase development in each system. Depending on the activating solution, the primary multi-gel phases that form in these types of geopolymers are sodium aluminosilicate hydrate (N-A-S-H), potassium aluminosilicate hydrate (K-A-S-H), calcium aluminosilicate hydrate (C-A-S-H), and calcium silicate hydrate (C-S-H), which are discussed in greater depth below. To strategically evaluate the complexity of the formed systems and to separately centralize the relevant information, the next subsections are individually focused on reaction developments and gel phases and permeability, as well as on shrinkage behavior.

#### 4.2.1. Reaction Developments and Gel Phases

Combining fly ash-based and slag-based geopolymers in one system produces more intricate gel products depending on the binders and liquids, which are used throughout the exothermic reaction. Using class-F fly ash as the main binder, and sodium or potassium hydroxide with/without sodium or potassium silicate as the alkaline liquid dominantly produces sodium aluminosilicate hydrate (N-A-S-H) gel or potassium aluminosilicate hydrate (K-A-S-H) gel. When slag is added, the system becomes more complex and could have multi-gel phases such as N-A-S-H, K-A-S-H, calcium aluminosilicate hydrate (C-A-S-H), and calcium silicate hydrate (C-S-H), and the formation of these gels differs depending on the raw materials used in the system.

**Figure 2:**
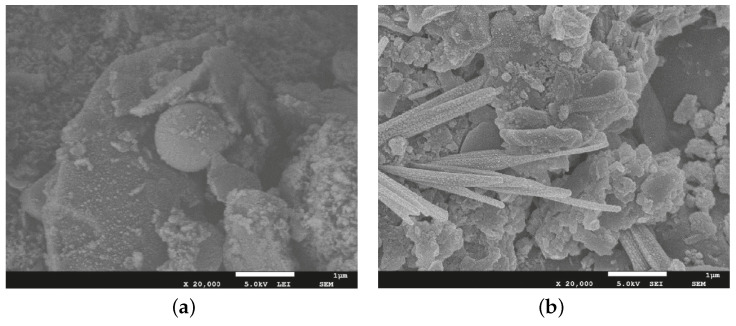
SEM images of the microstructure of samples with NaOH molarity of 8 M and (**a**) 100% fly ash and (**b**) 50% fly ash/50% slag [39].

Saha and Rajasekaran [26] evaluated the incorporation of GGBFS in class-F fly ash-based geopolymer. Scanning electron microscope (SEM) images were taken at the failure surfaces of compression-tested cubes. By incorporating more slag in 10% increments, the structure of the geopolymer paste was found to become denser due to an increase in calcium silicate hydrate gel (C-S-H), which consequently led to higher compressive strength measurements. Qiu et al. [39] studied the influence of the activator concentration and binder type on fly ash/blast furnace slag geopolymer as a mine backfilling material. The microstructure of the final products was composed of geopolymer gels or unreacted particles, either fly ash or slag particles. These unreacted particles appeared to be both fully or partially unreacted. With increasing fly ash amounts, the formation of the unreacted particles increased. Samples with 100% fly ash binder and 8 M concentrations revealed higher numbers of unreacted particles and lower density, as shown in Figure 2a. The amount of unreacted particles was reduced, and the matrix became denser as the slag content increased from 0% to 50% with the same molarity (see Figure 2b).

The fineness of fly ash or slag particles showed a notable impact on the mechanical properties and the formed final products. Tan and Pu [46] studied the combination of finely ground fly ash (FGFA) and finely ground granulated blast furnace slag (FGGBS) to evaluate its mechanical and microstructural properties. The researchers concluded that using FGFA and FGGBS as a combined system could achieve higher compressive strength and slightly improve the C-S-H gel formation, compared to FGFA and FGGBS when used independently. Jang et al. [25] evaluated the effect of superplasticizers on fresh and hardened slag/fly ash pastes to evaluate the setting time, mechanical strength, and microstructural characteristics. It was found that increasing the ratio of slag/binder from 0.3 to 1.0 led to a denser matrix in the hardened products. The addition of polycarboxylate-based and naphthalene-based superplasticizers up to 4% did not affect the final hydrated products, as shown by the SEM/EDS analysis. On the other hand, increasing the slag percentages in the binder produced cracks and a fast setting time when the slag ratio was 70% or higher. Puligilla and Mondal [47] evaluated the hardening products and the formation of phases in 1 h, 3 h, 24 h, and 14-day-old specimens using SEM in combination with energy-dispersive X-ray spectroscopy (EDS). The utilized binders were class-F fly ash and GGBFS, and the activators included potassium hydroxide and potassium silicate. Three hours after mixing, SEM analysis revealed that the specimens with no slag did not react because the fly ash spheres were clear, without any indication of a hydration product. Specimens with 15% of slag showed reaction products 3 h after mixing, and the gel evidently started to form. The EDS analysis for a sample at an age as early as 1 h showed K-A-S-H phase formation, whereas the samples with a 14-day age indicated the coexistence of K-A-S-H and C-A-S-H phases.

Based on the reported facts, it can generally be stated that the use of slag in fly-ash-based geopolymer increases reaction products at an early age and produces denser matrices, whereas the addition of superplasticizers shows minimal or no effect on the microstructural development in the fly ash/slag system. The number of unreacted or partially reacted fly ash particles decreases with increasing slag content. The coexistence of geopolymeric gels with calcium-rich phases such as C-A-S-H or C-S-H in the fly ash/slag system was proven to exist within one system. In addition to these potentially beneficial effects, it should be noted that adding excessive amounts of slag into the mixture may cause shrinkage, as further detailed in the following subsections.

#### 4.2.2. Permeability Properties

Permeability is an important characteristic of a hardened concrete mixture because it defines the susceptibility of the material to the ingression of aggressive solutions and its sensitivity to environmental effects. Permeability depends on the pore network and the microstructure of the concrete. The more permeable a concrete is, the more it is susceptible to aggressive gasses and liquids that may enter the material and potentially deteriorate the concrete matrix or the embedded reinforcing bars. This property has, therefore, been individually studied and the most significant reported results are discussed here to evaluate the effect of permeability on fly ash/slag-based systems.

**Figure 3:**
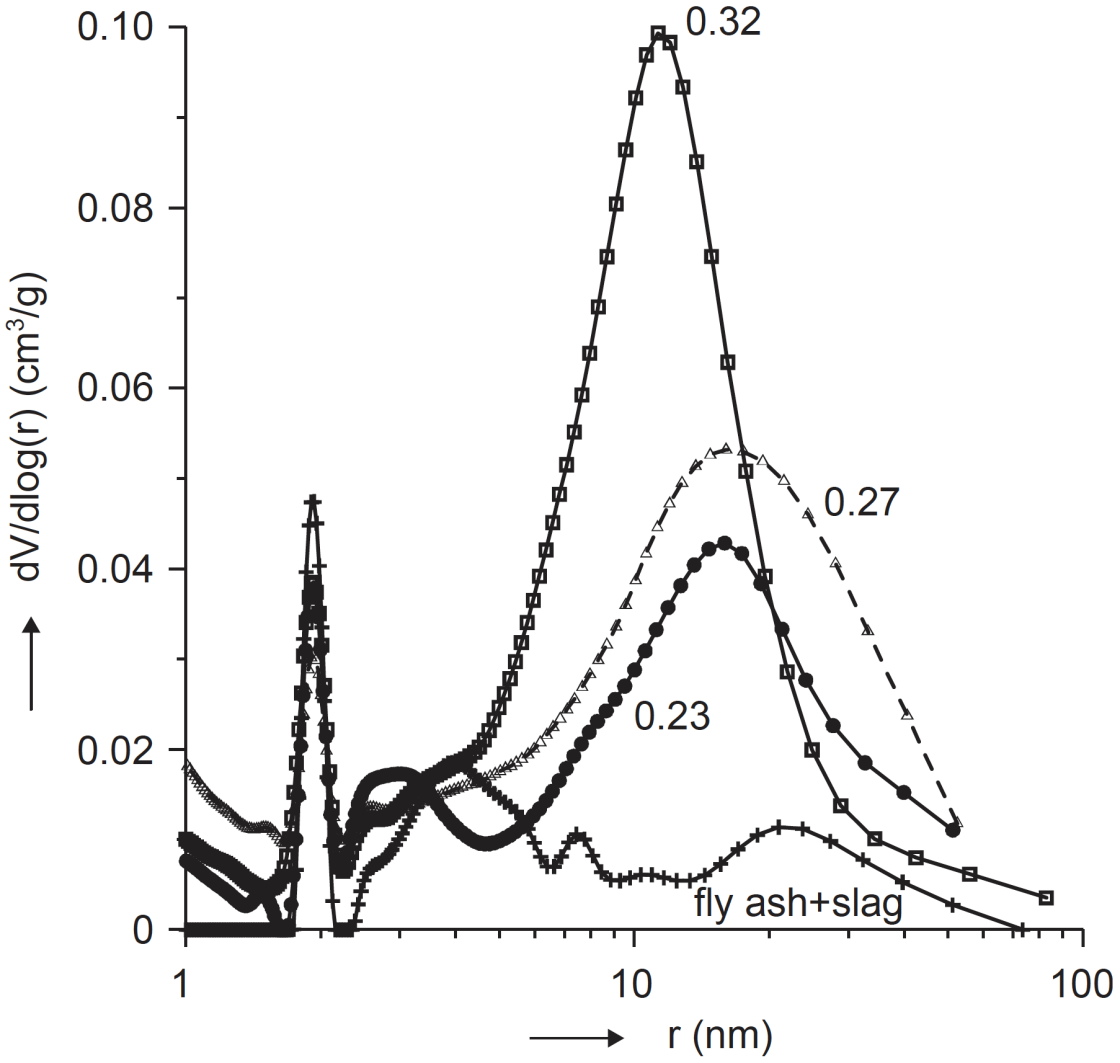
Pore size distribution of fly-ash-based geopolymer with water-to-fly-ash ratios of 0.23–0.32 and slag addition [23].

Ismail et al. [12] evaluated the volume of permeable voids (VPV) in accordance with [48] in fly ash/slag geopolymer mortar and concrete. The mortar porosity decreased slightly from 28 days to 90 days when the ratio of fly ash to slag was 0.7 or higher. In contrast, when the content of fly ash in the mixture reaches levels of 50% or below, the calcium aluminate silicate hydrate (C-A-S-H) gel dehydrates rapidly, inducing a more porous product at a 90 days age. In the case of concrete samples, the VPV of fly ash/slag geopolymer was higher than OPC concrete and decreased with an increase in the fly ash content. Deb et al. [28] studied the permeability and sorptivity of fly ash/slag geopolymers. The results demonstrated the effect of the addition of slag at 10% and 20% in comparison to the control group, which included fly ash only. The analysis showed a substantial reduction in permeability and sorptivity due to the incorporation of slag. The samples tested at 28 days that had only fly ash, 10% of slag, and 20% of slag revealed lower sorptivity and fewer permeable voids than regular OPC samples. Škvára et al. [23] studied the microstructural characteristics of fly ash-based geopolymers. The porosity analysis clearly showed that the addition of slag at 40% led to a reduction in porosity of 2% to 10% due to the coexistence of geopolymeric and C-S-H phases. As shown in Figure 3, the porosity was noticeably influenced by the water/fly ash ratio (water mass in the activator/fly ash mass) and slag addition, whereas the curing conditions had minimal effects on the permeability.

The higher the resistance to substance penetration into concrete, the lower the deterioration vulnerability for steel rebars and the concrete, depending on the aggressive and corrosive nature of the penetrating substances. Shang et al. [13] evaluated the chloride permeability of different combinations of fly ash and GGBFS geopolymers in reference to OPC and magnesium potassium phosphate cement (MKPC) mortars. Compared to OPC and MKPC mortars, the geopolymer mortars showed a general trend toward lower chloride diffusion coefficients. The decreasing trend in chloride diffusion coefficients was notably due to the increase in the GGBFS content in the fly ash geopolymer mortars. In this study, GGBFS mixtures showed higher strength values and denser matrices, which contributed to a gradual decrease in diffusivity. Because fly ash reacts slower than slag, it incrementally leads to lower permeability as the concrete matures. The coexistence of two or more gels in one system plays a significant role in permeability due to the interaction between these gels to produce a complex structural system. The hydration of the geopolymeric phase with or without Ca-rich phases produced denser structures and reduced the porosity more than that of the C-S-H phase formed primarily in OPC. The literature, therefore, reveals a general trend; fly ash/slag geopolymer systems show lower permeability than regular OPC, whereas increased slag volumes contribute to a further reduction in the permeability.

#### 4.2.3. Shrinkage Behavior

The shrinkage behavior of concrete is a crucial factor in the design of new mixtures because shrinkage may induce internal or external stresses that lead to significant cracks or microcracks, depending on the severity. Shrinkage of concrete can be induced at an early age or a later age and is commonly classified into four categories: plastic shrinkage, drying shrinkage, chemical shrinkage, and autogenous shrinkage. Plastic shrinkage is introduced to concrete soon after casting and before the setting of concrete due to the loss of moisture from the surface, whereas drying shrinkage occurs at a later age and is mostly introduced after the setting of concrete by drying environmental conditions, which eventually lead to moisture loss from the concrete. Chemical shrinkage is induced due to the difference in volume between the hydrated cement and unhydrated products of the total water and cement [49]. Autogenous shrinkage is a very minimal reduction in volume that occurs when water cannot migrate from or to the concrete, which is therefore mostly introduced under sealed conditions [49].

**Figure 4:**
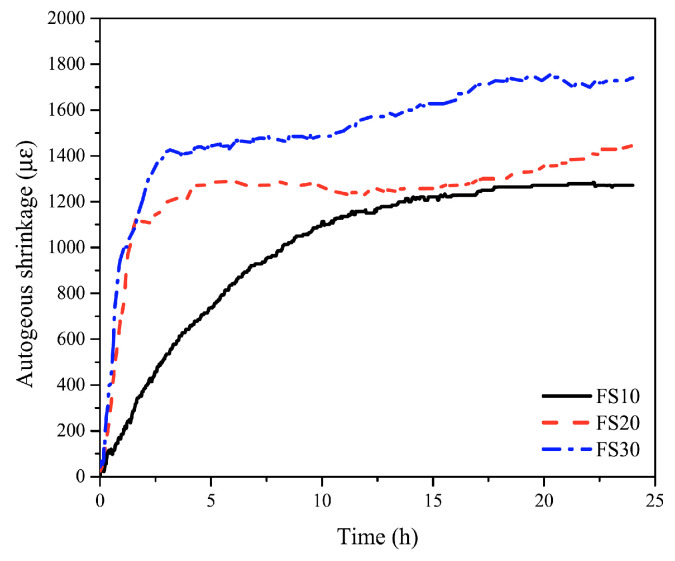
Autogenous shrinkage of AAFS pastes with 10%, 20%, and 30% of slag [29].

Fly-ash-based and slag-based geopolymers have different shrinkage characteristics, depending on the type of activator, and combining these systems leads to more complex hydration products and a more intricate shrinkage behavior [50]. Therefore, evaluating the aspects concerning shrinkage of fly-ash/slag-based geopolymer is essential in order to avoid unfavorable microcracks at early ages or significant cracks when the concrete is mature. Deb et al. [28] evaluated the drying shrinkage of slag and fly ash blended geopolymers. The drying shrinkage occurred up to a maturity of 56 days, in which all specimens (OPC and geopolymer concrete) measured values below 650 microstrains. It was noted that incorporating 10% and 20% of slag by weight reduced the shrinkage by 20% and 50%, respectively. A study by Shang et al. [13] was conducted to determine the drying shrinkage for different combinations of FA and GGBFS geopolymers in references to companion OPC and MKPC mortars. The evaluation of drying shrinkage revealed a decrease in the performance of geopolymers in comparison to OPC and MKPC mortars. The volume stability of these geopolymers showed no correlation to curing time; however, the higher content of GGBFS had a negative influence on the shrinkage. The study concluded that less than 20% of GGBFS as a partial replacement for fly ash resulted in a similar OPC mortar performance. Fang et al. [29] evaluated autogenous and chemical shrinkage at a maturity of 24 h for alkali-activated fly ash-slag (AAFS) by replacing slag with fly ash at levels of 10%, 20%, and 30% by weight. It was found that AAFS pastes with the three different slag contents had similar trends, with different values of autogenous shrinkage (see Figure 4).

When increasing the level of slag replacement from 10% to 30%, the ultimate autogenous shrinkage rose from 1271 μ” to 1740 μ”. The highest autogenous shrinkage rate was recored to be 942 μ”/h for 30% of slag replacement after only 1 h of casting, which was followed by 696 μ”/h at 1h age and 201 μ”/h at 2 h age for 20% and 10% of slag replacements, respectively. The shift in outrageous shrinkage resulting from higher slag replacements could be attributed to the change in the capillary pressure. The increase in slag content increased the densification of the reaction products, which increased the capillary pressure and resulted in autogenous shrinkage [51]. For the chemical shrinkage of AAFS pastes, ultimate shrinkage rose from 0.018 $mLg^{-1}$–0.021 $mLg^{-1}$ when slag contents increased from 10% to 30%, which resulted in a higher volume reduction of the final reacted products. Increasing the slag content accelerated the alkali reaction rate and contributed to a higher volume reduction when compared to the unreacted paste [29].

Castel et al. [52] evaluated various temperatures and durations for the heat curing of blended fly ash and GGBFS geopolymer. It was found that curing at 40 °C for one day was not sufficient to meet Eurocode 2 requirements for shrinkage. The study concluded that at least three days of 40 °C and one day of 80 °C heat curing are sufficient to meet such shrinkage requirements. Lee and Lee [31] evaluated the mechanical and setting developments geopolymers with combinations of fly ash and slag at 0.1–0.3 ratios (slag/fly ash) by weight. It was found that the compressive strength was unstable at a replacement of 30% slag, and that surface cracks developed, potentially due to shrinkage. Specimens with 10%, 15%, 20%, and 25% of slag were more stable, and did not develop cracks. Singh et al. [53] evaluated the drying shrinkage of fly ash/slag geopolymer concrete with varying NaOH concentrations from 10 M to 16 M and a ratio of 2.5 of sodium silicate-to-sodium hydroxide. Drying shrinkage was measured at specific time intervals for six months. The ultimate shrinkage was achieved at a maturity of 180 days (the end of the study period), which was reported with 1045 μ”. It was noted that the drying shrinkage of fly ash/slag geopolymer attained almost 89% of the ultimate value for the specimens at 28 days age. Throughout the six months of monitoring, a significant increase in shrinkage was noted throughout the first seven days.

The primary trend from the previously conducted studies that were reviewed for this paper revealed that the addition of slag and its quantity significantly contribute to the shrinkage behavior of geopolymers. Excessive slag contents in the mixture may lead to higher and undesirable shrinkage values. Drying, autogenous, and chemical shrinkage increase under elevated slag contents, as shown by various studies. The rises in the shrinkage values may promote the development of micro- or major cracks. In addition, curing temperature and curing durations influence shrinkage behavior. Finally, it is clear that the optimum curing conditions and slag content replacement have to be properly determined to avoid undesirable shrinkage behavior.

### 4.3. Durability Properties

Permeability plays a primary role in the durability of slag/fly ash geopolymers because higher permeability characteristics allow the penetration of aggressive ions, which expedite the degradation process. The resistance to severe environments decreases with increasing concrete permeability, due to the penetration of substances towards the steel reinforcement. Compressive strength, tensile strength, and porosity are all negatively affected when a geopolymer is exposed to severe environments [54]. Similarly to ordinary Portland cement concrete, environmental conditions such as chloride ingress, freeze-thaw cycles, sulfate attack, acid exposure, and carbonatation are common environmental factors that may affect geopolymer performance. The following subsections separately detail the findings for resistance against chloride ingress, sulfate attack, and carbonatation of fly ash/slag geopolymer to better target each individual aspect and because these are common durability parameters that describe either the degradation process or the decrease in overall performance of geopolymer concrete.

#### 4.3.1. Chloride Resistance

Chlorides are the most dominant components in a saline environment that lead to the disintegration of reinforced concrete. Chloride attack is referred to as one of the main concerns in relation to the durability of concrete due to its corrosive effect on the reinforcing steel bars after penetrating the concrete. Noushini et al. [55] evaluated the migration and diffusion coefficients and the binding capacity of chloride in low-calcium fly-ash geopolymer with the partial substitution of slag. Chloride binding is the difference between free and bound chloride ions to concrete at different concentrations and fixed temperatures [56]. For this study, the chosen chloride immersion duration was relatively short (with 35 days), whereas a relatively high concentration of aqueous sodium chloride solution with 16.5% (165 g of NaCl in 1 L solution) was used. A total of 12 different curing conditions were used, focusing on three temperatures and four curing periods. Increasing the curing duration and temperature resulted in lower migration coefficients. Although the pore structure played a significant role in chloride diffusion coefficients, the chloride binding capacity showed a minimal effect on chloride diffusion coefficients. The study concluded that low-calcium fly ash was not the optimum option for rich chloride environments due to its low resistance to chloride diffusion and binding.

**Figure 5:**
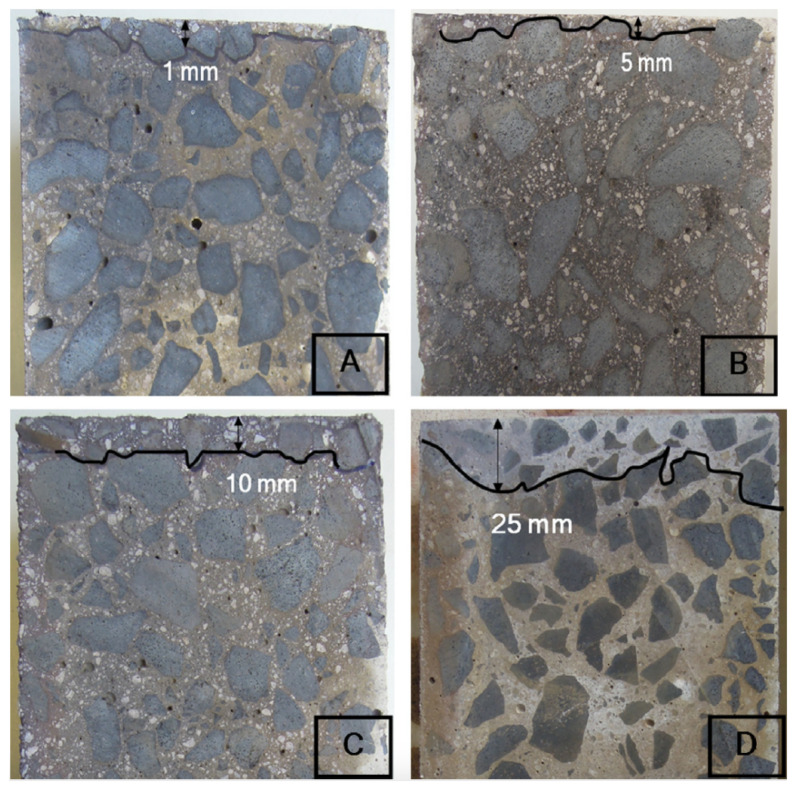
Chloride penetration depth using ponding test for concrete at 28 days for (**A**) 100/0, (**B**) 75/25, (**C**) 50/50 (wt.% slag/ wt.% fly ash), and (**D**) OPC concrete (Reprinted with permission from Ref. [12]. Copyright 2013 Elsevier).

The ASTM-International [57] and Nordtest [58] standards offer a methodology for chloride resistance testing. Ismail et al. [12] used the NordTest NT Build 492 [58] standard and ASTM-International [57] with a slight modification to assess the penetration of chloride. Class-F fly ash and granulated blast furnace slag were used to prepare geopolymer concrete and mortar specimens with different fly ash/slag ratios. The mortar samples were demolded 24 h after casting, before they were sealed and placed in an oven at 30 °C until the testing day. After the concrete samples were demolded (also 24 h after casting), they were placed in a water bath at ambient temperature until testing was initiated. The chloride penetration depths were significantly affected by the slag/fly ash ratio and by the maturity of the final products that were formed. Figure 5 demonstrates that the chloride penetration depth increased as the fly ash substitution increased.

However, the penetration depths were substantially reduced at later maturities (90 days), as for slag-to-fly ash ratios of 100/0, 75/25, and 50/50 (wt.%) the measurements were 0, 0, and 1 mm, respectively. The chloride penetration depth in OPC concrete decreased from 25 mm at 28 days to 20 mm at 90 days.

Zhu et al. [10] examined the porosity and tortuosity of chloride penetration in fly ash/slag-based geopolymer pastes and mortars. Tortuosity was defined by Bear [59] as the ratio of the real flow path relative to the straight line from the start to the end of the flow path. Samples followed three temperature curing stages at 20 °C for 1 day, 65 °C for 2 days, and 20 °C for 14 days; the relative humidity (RH) was fixed at 95%±5%. Without using slag in the pastes and with increasing liquid/binder ratios, the chloride penetration increased. With 40% of fly ash replacement by slag, the chloride penetration was reduced significantly and fell under the levels measured for OPC pastes. Additionally, when 40% of slag was added, the porosity of the final product did not change; however, the tortuosity increased, which led to a substantial reduction in chloride penetration. These findings suggest that chloride penetration is related not only to the porosity but also to the tortuosity of the pore structure, which may be the main factor. Rajagopalan [60] studied the durability of low-calcium fly ash/slag blends in an accelerated test for corrosion in NaCl solution. The researchers followed the “Florida Method of Test for an accelerated Laboratory Method for Corrosion Testing of Reinforced Concrete Using Impressed Current (FM 5-522-2000)” [61]. This test method was applied to geopolymer samples with GGBS as the primary binder, which was substituted with fly ash by up to 50%. When slag was partially replaced by 10% to 30% and 50%, the crack initiation duration ranged between 18 and 22 days. However, the replacement of slag with 40% of fly ash showed an improvement in the resistance of chloride ion penetration, and the crack initiation duration started after 32 days. For chloride penetration testing, Yang et al. [62] used chloride diffusion and X-ray fluorescence (XRF) to measure the concentration of chloride in fly ash/slag geopolymers after the specimens were pulverized. The research concluded that the inclusion of 50% of slag content attained higher resistance to chloride penetration due to the improvement of pore structure and concrete sorptivity.

These studies show that chloride migration in fly ash/slag geopolymer is primarily affected by the fly ash content and the maturity of the formed reaction products. Although increasing the fly ash content significantly worsens the chloride penetration, increased curing durations and temperatures evidently improves chloride resistance. Accordingly, it is recommended to use 40% or more slag substitution in chloride-rich environments [10,60]. An increased slag content improves the pore structure of geopolymers in fly ash/slag blends, which significantly affects the resistance to chloride penetration.

#### 4.3.2. Sulfate Attack

Sulfate attack on concrete mainly occurs when sulfate ions react with concrete compounds such as calcium hydroxide Ca(OH)_2_ and calcium aluminate hydrate. This reaction may cause deterioration or concrete expansion due to the formation of gypsum and/or ettringite. Ettringite formation is considered to be one of the most destructive reaction products that harms concrete. This reaction starts with the ingression of sulfate ions into the binder constituents of concrete, where they react with calcium-rich phases (C-S-H or C-A-S-H) to form calcium sulfate (CaSO_4_). After the formation of calcium sulfate (gypsum), it reacts with tricalcium aluminates to produce calcium sulfoaluminate, which is also known as ettringite [63]. A concrete structure may encounter sulfates when exposed to environments such as seawater, groundwater, or sulfate-rich soil. However, sulfate attack in geopolymer components may result not only from external environments, but also from substances within the mixture [64].

Karthik et al. [65] studied the durability of fly ash/slag (60/40) geopolymer combinations with the addition of bio-additives (*Terminalia chebula* and natural sugar). Samples were immersed in sodium sulfate solution (Na_2_SO_4_) with a 5% concentration. Specimens without bio-additives suffered significantly more density losses, with a 13.97% density loss compared to a 3.91% density loss for the addition of bio-additives, and the compressive strength loss was measured up to 2.95%. Bio-additives decreased the permeability of fly ash/slag samples compared to the samples that were free from bio-additives. In a study conducted by Gopalakrishnan and Chinnaraju [40], different fly ash/slag combinations were tested at fly ash levels from 0% to 50% and slag levels from 50% to 100%. The study was conducted over 180 days, with an immersion in a 5% concentration of Na_2_SO_4_ and MgSO_4_ solutions, which were both renewed every 30 days. A general trend in strength loss was noted, which was correlated to the increased fly ash replacement. Replacing slag with 40% of fly ash revealed the highest performance among the evaluated mixes and it caused no formation of gypsum or ettringite under sulfate environments. All samples gained weight after immersion for 180 days; the samples immersed in sodium sulfate gained weight ranging between 0.45% and 0.972%, whereas the weight gains for the samples immersed in magnesium sulfate ranged between 0.585% and 0.730%.

**Figure 6:**
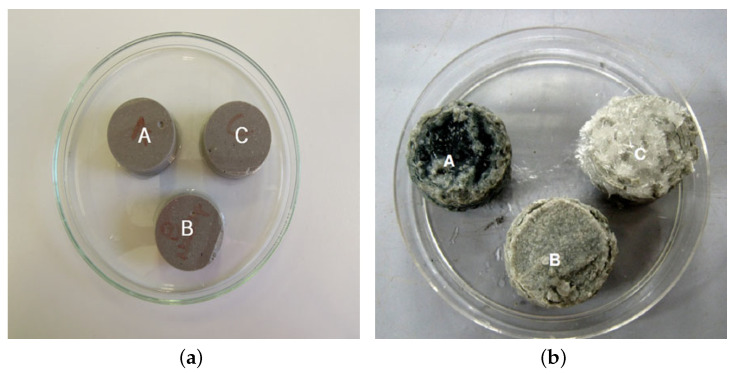
External attacks from sodium (**a**) and magnesium (**b**) sulfates on slag/fly ash geopolymer samples (A: w/b 0.4, B: w/b 0.5, and C: w/b 0.6) (Reprinted with permission from Ref. [66]. Copyright 2012 Springer Nature).

Ismail et al. [66] evaluated changes in the microstructure of fly ash/GGBFS geopolymers with a 1:1 ratio under sodium and magnesium sulfate exposure. Samples were cured for 28 days, and then separately stored (for an additional 90 days) in capped containers, which were filled with the specified solutions. Magnesium sulfate was more aggressive to the geopolymer structure and caused decalcification of the calcium-rich phase and gypsum formation, whereas sodium sulfate had minimal effects on the geopolymer system. The activator-to-binder ratio (w/b) played a significant role in the pore structure and the resistance to the sulfate attack. Figure 6a,b shows the effect of sodium and magnesium sulfates with different ratios of w/b from that study.

Figure 6a shows that there were no effects of sodium sulfate on any of the samples for all the evaluated ratios, whereas the magnesium attacks shown in Figure 6b revealed higher gypsum formation, which can be seen in sample c, with a w/b ratio of 0.6. Džunuzović et al. [67] evaluated the effects of sodium sulfate attack on slag/fly ash geopolymer mortars. The specimens were cured for 28 days and then exposed to environments that led to sulfate attacks. Assessments were performed after 30, 90, and 180 days of exposure. The authors concluded that Na_2_SO_4_ solution did not affect the mechanical strength, nor did it cause a formation of new phases in fly ash/slag-based geopolymers; however, leaching of elements such as Si, Ca, and Na was noted. The external sodium sulfate environment induced the slow development of the compressive strength but did not affect the final development of compressive strength at 180 days.

In the literature, the focus thus far appears to be on sodium and magnesium sulfate solutions to study the resistance of fly ash/slag geopolymer systems against sulfate attacks. Different solution concentrations and immersion durations have been evaluated. In most cases, sodium sulfate generally showed a minimal or positive effect on the fly ash/slag geopolymer blends. On the other hand, magnesium sulfate attack led to the formation of gypsum, which consequentially causes a reduction in mechanical strength properties. Accordingly, it is clear that geopolymers based on fly ash and slag as raw materials are influenced by the fly ash dosage when under sulfate attack. Similarly to the observations made for chloride attacks, increasing fly ash amounts contributed to a reduction in sulfate resistance. Table 2 summarizes the developments in this area regarding fly ash/slag geopolymers, listing the important findings and different synthesis conditions for sulfate environments.

#### 4.3.3. Carbonatation

Carbonatation of concrete is a critical phenomenon for the quantification of concrete durability because it may lead to changes in the microstructure and moisture content, which both affect the porosity, pore connectivity, pore size distribution, and water release, which subsequently may affect the permeability and diffusion coefficients of concrete [68]. Carbonatation is engendered naturally when carbon dioxide (CO_2_) from the atmosphere directly penetrates material and is absorbed by the concrete surface [69]. After the penetration, CO_2_ reacts with calcium silicate hydrate (C-S-H) and calcium hydroxide (Ca(OH)_2_) to produce H_2_O and calcium carbonate (CaCO_3_) [70].

**Figure 7:**
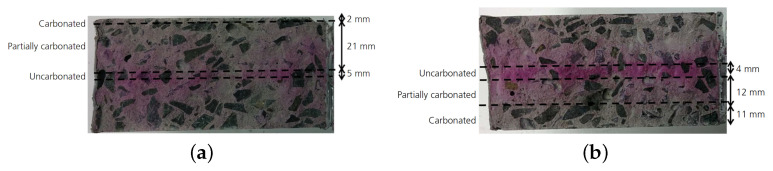
Carbonation depth for samples exposed to (**a**) 1% concentration of carbon dioxide after 2 weeks and (**b**) 3% concentration of carbon dioxide after 6 weeks (reproduced from [71]; copyright 2017 ICE Publishing).

Pasupathy et al. [69] studied the carbonatation of fly ash and slag geopolymer specimens that were aged for eight years. The specimens were extracted from the cores of two types of slab, located adjacent to each other under ambient atmosphere. The first type was classified as type I and was composed of 75% fly ash/25% slag, whereas type II was composed of 70% fly ash/30% slag. Phenolphthalein indicators were used to measure the depth of carbonatation. The study’s authors came to the conclusion that 75% fly ash/25% slag geopolymer specimens demonstrated much lower resistance to carbonatation, at 23.5 mm to 27.5 mm, whereas samples with 70% fly ash/30% slag significantly improved from (8 mm to 14 mm) and showed similar tolerance to carbonatation to that of ordinary Portland cement (7 mm to 13 mm). In a study conducted by Khan et al. [71], low-calcium fly ash and ground granulated blast furnace slag were used as an aluminosilicate source with 90% fly ash and 10% slag. Samples were first cured at 75 °C for 18 h and then demolded, before they were left in a chamber that maintained a constant temperature of 23 °C ± 2 °C and a relative humidity of 55% until testing. Samples were cut into 50 mm-segments and sealed to leave only the top and bottom of the specimens exposed to carbon dioxide. The exposure duration ranged between two and six weeks, except for the samples exposed to natural carbonatation, for which the duration was extended to six and 18 months. For samples exposed to an environment with 1% accelerated carbonatation, natron formed after six weeks of exposure. When the concentration of carbon dioxide increased to 3%, nahcolite formed after only two weeks of exposure. Figure 7 depicts the carbonatation depth of the samples exposed to 1% and 3% concentration of carbon dioxide.

**Figure 8:**
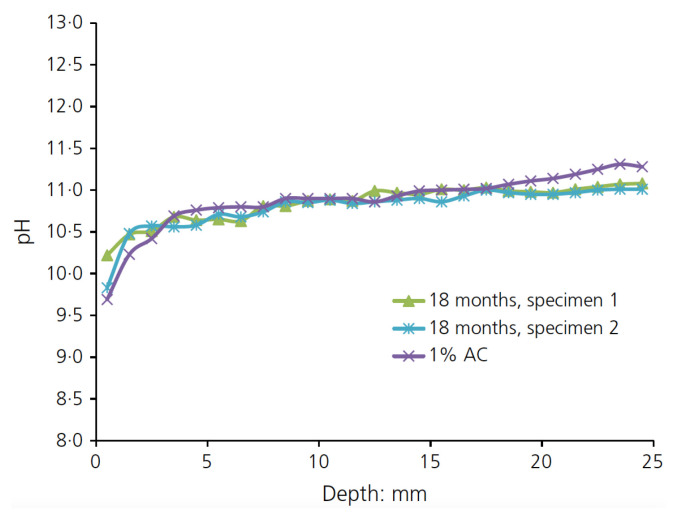
pH values for eighteen months of exposure to natural carbonatation and 1% of accelerated carbonatation for 6 weeks (reproduced from [71]; copyright 2017 ICE Publishing).

For samples exposed to 1% of carbon dioxide for six weeks, the depth of carbonated segments reached 2 mm, whereas a level of 3% carbon dioxide at a shorter exposure duration of two weeks led to a significant increase in penetration depth (up to 11 mm). The measurements of pH values and carbonatation depths of samples that were exposed for 18 months to natural carbonatation were almost identical to the measurements for samples exposed to 1% of accelerated carbonatation for six weeks, as shown in Figure 8.

**Figure 9:**
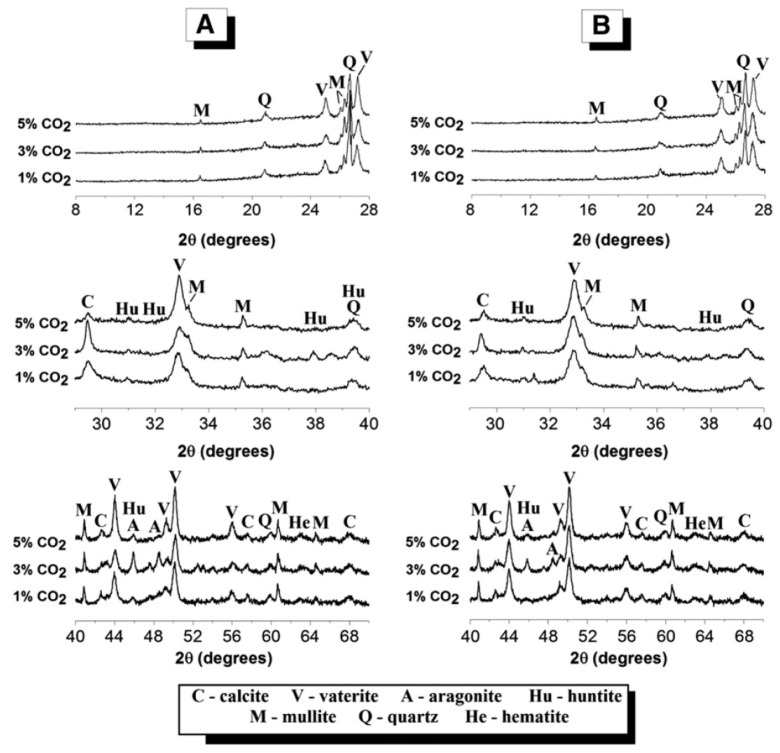
X-ray diffractograms of blended fly ash and slag after (**A**) 1 day and (**B**) 7 days of accelerated exposure to three concentrations of carbon dioxide—1%, 3%, and 5% (Reprinted with permission from Ref. [73]. Copyright 2013 Elsevier).

Zhuguo and Sha [72] evaluated the effect of carbonatation on geopolymers made from fly ash and granulated blast furnace slag, with a focus on different parameters such as NaOH content, fly ash/slag ratio, slag fineness, heat curing, and retarder usage. When the slag content increased from 20% to 50% in the mixture, the carbonatation resistance increased. Higher fineness of slag and elevated curing temperatures revealed higher resistance to carbonatation. Samples cured at 60 °C and 80 °C displayed an increased resistance to carbonatation by a factor of three in comparison to the samples cured at 20 °C. The ratio of Na/Si in sodium silicate and sodium hydroxide affected the carbonatation rate as well, as an increased Na/Si ratio led to a reduction in carbonatation resistance. The evaluated retarder was an inorganic compound and was added at 5% which caused a slight decrease in the carbonatation coefficient, compared to the samples that were prepared without any retarder. Bernal et al. [73] studied the effects of accelerated carbonatation on fly ash/slag geopolymer binders, which were based on class-F fly ash and granulated blast furnace slag. Fly ash and slag were mixed at a 1:1 ratio, before the resulting geopolymer samples were cured at a constant temperature of 23 °C. After curing for 1 and 7 days, the samples were crushed in a controlled carbon dioxide environment for accelerated carbonatation. Three concentrations of carbon dioxide of 1%, 3%, and 5% were examined at a temperature of 23 °C ± 2 °C and a relative humidity of 65% ± 5%. The accelerated carbonatation after 1 and 7 days of exposure led to the formation of huntite and three crystalline polymorphs of calcium carbonate (calcite, aragonite, and vaterite). Huntite formation increased in the 3% group, as compared to the group with 5% of CO_2_ concentration, and its level was found to be higher in the less developed gel (at 1 day) than in the mature gel (at 7 days), as exemplified in Figure 9.

Previous studies show that carbonatation resistance is mostly affected by carbon dioxide concentration, exposure duration, curing conditions, and the proportions of raw materials. Similarly to the findings made for chloride and sulfate attacks, carbonatation resistance increases as the slag content is magnified. Substituting fly ash with slag by 30% or more appears to be effective as it improves carbonatation resistance. Furthermore, initially curing geopolymers at elevated temperatures cause a more consistent formation of reaction products, which led to additional resistance against carbonatation.

## 5. Discussion

Low-calcium fly-ash-based geopolymer has a slow setting time [24,25,35] and requires heat curing to accelerate the hardening process [25,41]. Adding slag, on the contrary, leads to a more rapid setting time [25,29,42]. Combining these two binders revealed positive effects on setting time, mechanical strength, permeability, and durability in aggressive environments. Significant changes in the fresh properties and the microstructure formation can be traced back to the addition of slag, which in turn affects the hardened properties of the fly-ash-slag geopolymer; specifically, characteristics such as setting time, gel phases, matrix density, shrinkage, and permeability are improved in the binary system. In an effort to correlate the various material characteristics, an extensive search through publications in the field of fly-ash-slag geopolymer systems was conducted and a comprehensive review was summarized above, and we further synthesize and discuss the findings and identified trends below.

Based on the rapid setting time induced through the use of slag, it now appears to be clear that the high calcium composition from slag reacts rapidly with the available silica offered by sodium silicate in the alkaline solution. Numerous studies used sodium silicate and sodium hydroxide as the alkaline activator, and the setting time decreases in most cases when the ratio of sodium silicate to sodium hydroxide increases [24,32]. This is due to the higher amount of soluble silica offered by the alkaline solution, which accelerates the rate of the setting time and contributes to the final gels formed [32,42]. The Ca/Si ratio in the formed C-S-H gel in slag-based geopolymer is lower than that formed in ordinary Portland cement, and by introducing class-F fly ash into the reaction, the Ca/Si ratio is made substantially smaller than what is normally formed in OPC. The decrease in the Ca/Si ratio could be attributed to the substitution of Al^+3^ for Si^+4^ in the calcium-rich phase [74]. Although the addition of slag to fly-ash-based geopolymer significantly accelerates the setting time, increases mechanical strength, and densifies the final gels, it may also negatively contribute to volume instability [13,29] and crack initiation [31]. The literature has shown that shrinkage of the hardened system as a result of a larger substitution of fly ash with slag may lead to crack propagation. Accordingly, it appears to be crucial to determine the proper replacement ratio limitations to optimize the desired characteristics in the combined system.

The multi-gel phases formed within the fly ash-slag system produce a more intricate and denser microstructure, which ultimately improves the permeability. Permeability is an essential material property because it controls the flow paths for aggressive solutions that penetrate and harm the concrete. The literature clearly demonstrate that the coexistence of geopolymeric gel phases (N-A-S-H or K-A-S-H) and calcium-rich gel phases leads to a substantial reduction in permeability. When these gels form in one system, they produce a denser microstructure, which eventually contributes to low permeability. Because this study focused on durability concerning chloride ingress, sulfate attack, and carbonatation, permeability is discussed in the context of these aspects. For chloride environments, different standards designed to evaluate the durability of OPC systems—including NordTest NT Build 492, ASTM-International [57] (withdrawn in 2019), and ASTM-International [75]—have been utilized to analyze the ingress of chloride into fly ash/slag geopolymers. Among the factors that contribute to chloride ingression, the fly-ash-to-slag ratios and the maturity of the reaction products substantially affect the chloride penetration depths. According to the literature about chloride attacks on fly ash/slag-based geopolymers, the consensus is that chloride resistance is affected by the curing duration [55], curing temperature [55], pore structure [10,55,62], fly ash/slag ratio [10,12,60,62], liquid/binder ratio [10], and the maturity of the formed products [12]. Furthermore, porosity and tortuosity are both significant factors in chloride resistance [10]. An important finding by Zhu et al. [10] demonstrated that Cl^−^ penetration not only relates to porosity but also to the tortuosity of the formed structure. Thus, a reduction in the porosity and an increased tortuosity lead to an improved resistance to Cl^−^ migration. The inclusion of slag improves the pore structure system, such as the porosity and tortuosity, which leads to low permeability and increases the chloride penetration resistance.

As permeability plays a significant role in chloride ingression, sulfate attack and carbonatation are also affected by the pore structure of fly ash/slag-based geopolymer systems. Sulfate ions penetrate fly ash/slag geopolymer and cause the formation of gypsum and/or ettringite, which contribute to the expansion or contraction of the affected concrete element. Expansion or contraction induces micro- and major cracks, which ultimately may cause the disintegration of the whole structure. The ingression of sodium sulfate solutions in fly ash/slag geopolymers is less aggressive on the formed microstructure and some studies have identified negligible or positive gains in the mechanical strength [66,67]. On the other hand, magnesium sulfate solution contributes to gypsum and ettringite formation, which induced the expansion and deterioration of the structure [40,66]. For carbonatation, adding slag to the fly-ash-based geopolymer system positively affects the carbonatation resistance [69,72]. The decrease in the apparent volume of permeable voids (AVPV) resulting from higher substitution of slag shows a positive effect and a strong correlation between carbonatation resistance and AVPV [69]. Gel phases formed after the addition of slag are denser, and therefore decrease the permeable voids through which CO_2_ travels to penetrate the matrix. The reaction products formed throughout this process produce natron, huntite, and calcium carbonate, which may lead to the decomposition of the hydrated products [71,73]. Although slag affects the corbonatation behavior as described above, other factors such as exposure duration, carbon dioxide concentration, and curing conditions significantly impact the carbonatation process. Evaluating material parameters that induce internal or external changes in the sulfate environment or carbonatation process is critical when designing fly ash/slag geopolymers.

Fly ash/slag-based geopolymer properties vary according to the fly ash/slag ratio, liquid/binder ratio, available soluble silica, pore structure, and curing conditions and duration. The formation of C-S-H or C-A-S-H gel phases in fly-ash-based geopolymers leads to low permeability, which in turn increases the resistance to aggressive solutions that may deteriorate the reinforcement steel or the concrete matrix. This reduction in permeability increases durability in aggressive environments (sulfate or chloride ions), as well as the resistance to carbonatation in carbon-rich environments. However, it should be noted that the replacement quantity of slag should be constrained to limit crack initiation due to shrinkage susceptibility. The addition of slag to low-calcium fly ash-based geopolymer densifies the microstructural system and increases the uptake of calcium-substituting alkalis, which leads to C-A-S-H formation [76]. Substituting alkalis for calcium due to the higher affinity of calcium to silicon, which leads to the formation of C-A-S-H, may delay the hydration of N-A-S-H or K-A-S-H [77]. Delaying the formation of N-A-S-H or K-A-S-H may lead to lower strength gains and delay the ultimate strength of these materials at later ages.

The interactions and mechanisms of calcium-rich phases (C-S-H and C-A-S-H) and N-A-S-H or K-A-S-H are complex due to the multiple factors involved in the reactions that mainly include alkaline solution materials, the chemical compositions of fly ash and slag, the concentrations and proportions of materials in the alkaline solution, the ratios of fly ash/slag and alkaline activator/binder. Despite previous extensive studies, in which researchers have attempted to evaluate pore structure [12,28], gel phase formations and structures [47,76,78], elemental compositions [78,79], and microstructural density [26,39], the exact mechanisms underlying the interaction between fly ash/slag blended systems and alkaline activators remains unclear.

## 6. Conclusions

In an effort to centralize the important data from various studies with a focus on low-calcium fly ash/slag geopolymers, numerous research papers were reviewed and summarized. The general trends found in the literature were detailed, summarized, and comprehensively discussed. Based on an extensive analysis of different parameters, such as variations in material proportions (fly ash/slag, liquid/binder), curing conditions, curing duration, and supplemental additives, correlations between these parameters were identified, which led to the following conclusions:

- The setting times of fly ash/slag-based systems are affected by multiple factors that include the fly ash/slag ratio, the type and amount of superplasticizers, activator concentration, and available soluble silica. Among these factors, the fly ash/slag ratio and the available silica content in the activator are the dominant parameters that impact the setting time. However, polycarboxylate-based superplasticizers are effective when used for the evaluated combined systems and effectively retard the initial and final setting time.
- Incorporating higher slag quantities increases the microstructure density and reduces the number of unreacted particles due to the formation of calcium-rich phases that include C-S-H (similar reaction products to those found in OPC systems) and C-A-S-H (the main reaction product in slag-based geopolymers, with a higher aluminum content).
- The coexistence of geopolymeric and calcium-rich gel phases results in substantially reduced permeability, and substituting high percentages or excessive amounts of slag may lead to shrinkage and produce micro-cracks.
- The ratio of fly ash to slag and the maturity of the reaction products substantially affect the chloride penetration depths due to the change in permeable voids. However, chloride penetration does not only depend on the amount of pores as the permeability is also affected by the pore geometry (tortuosity).
- For fly ash/slag geopolymers, the ingression of magnesium sulfate ions is more aggressive than that of sodium sulfate ions. Fly ash/slag-based systems in magnesium sulfate environments form gypsum and/or ettringite products, whereas the effect of sodium sulfates is negligible or positively affects these binary geopolymer systems.
- Curing conditions and the gel maturity of fly ash/slag-based geopolymer are the dominant factors that determine the carbonatation resistance, which needs to be properly controlled because the carbonatation of fly ash/sag systems may form undesirable compounds such as natron, huntite, and calcium carbonate.
- The interaction of low-calcium fly ash and slag with an alkaline solution produces multiple phases and introduces complex mechanisms (multi-gel phase structures, elemental compositions, pore structures) that are still not fully understood and require further investigation.

## Figures and Tables

**Table 1:** Influential parameters on setting time.

Reference	GGBFS ^*^	(SS/SH) ^**^	Molarity	Notes
[31]	10–30%	0.5, 1.0, and 1.5	4 M, 6 M, and 8 M	Alkaline-solution-to-binder ratio was 0.38
	Setting time decreased with the increase in GGBFS	Fastest setting time when SS/SH was 1.5. For 4 M, increasing the SS/SH ratio decreased the setting time	When increasing the molarity, setting time decreased	Phosphoric acid (H_3_PO_4_) was used with 0.5%, 1.0%, 1.5%, 2.0%, and 2.25% (by weight) of the total binder
		For 6 M, a 1.0 ratio revealed the longest setting time		Initial setting time with 0.5–2.0% (H_3_PO_4_) slightly increased, whereas final setting decreased
				For 2.25%, the initial and final setting times increased
[32]	10–30%	1.5, 2.0, and 2.5	14 M	Alkaline-solution-to binder ratios were 0.35, 0.40, and 0.45
	Setting time decreased with the increase in GGBFS	When increasing the ratio of SS/SH, the setting time decreased		Setting time increased with the increase of the solution/binder ratios
[24]	0–40%	1.0, 1.5, 2.0, and 2.5	14 M	Added free water with ratios of 0.09, 0.12, and 0.15 (free water/binder)
	Setting time decreased with the increase in GGBFS	When increasing the ratio of SS/SH, the setting time decreased		Alkaline-solution-to-binder ratios were 0.4, 0.5, 0.6, and 0.7
				Setting time increased with the increase of the solution/binder ratio and the relationship between them was almost linear
[25]	0–100%	SS/SH = 2	4 M	Alkaline-solution-to-binder ratio was 0.5
	Setting time decreased with the increase in GGBFS			Polycarboxylate-based and naphthalene-based superplasticizers were used
				Initial and final setting time increased by 50 min and 70 min by adding polycarboxylate-based superplasticizer
[35]	0–50%	No sodium silicate was used	6 M	Alkaline-solution-to-binder ratio was 0.35
	Setting time decreased with the increase in GGBFS			
[26]	0–50%	SS/SH = 1	6 M, 8 M, 10 M, 12 M, 14 M, and 16 M	Alkaline-solution-to-binder ratio was 0.4
	Setting time decreased with the increase in GGBFS		With increasing the molarity, setting time decreased	
[29]	10–30%	SS/SH = 2	10 M	Alkaline-solution-to-binder ratio was 0.4
	Setting time decreased with the increase in GGBFS			Polycarboxylate based superplasticizer was used with the mass ratio of 0.01 to binder

[*] GGBFS: ground granulated blast furnace slag. [**] SS/SH: sodium silicate/sodium hydroxide.

**Table 2:** Sulfate attack literature.

Reference	Binders and activators	Conditions	Notes
[67]	Class F-FA^*^ (50%)	Duration: 180 days	Leaching of Na, Si, and Ca in Na_2_SiO_3_, but did not cause significant instability in the structure
	GGBFS (50%)	5% Na_2_SO_4_ solution	Slight increase in compressive strength in sodium sulfate environment
	Na_2_SiO_3_ modified by NaOH solids	Renewed every 30 days	The ratio of Si/Al decreased due to the leaching of silicon
[66]	Class F-FA (50%)	Duration: 90 days	Magnesium sulfate was more aggressive than sodium sulfate
	GGBFS (50%)	5% Na_2_SO_4_ solution	The immersion in magnesium sulfate caused decalcification of the phases containing Ca
	Na_2_SiO_3_	5% MgSO_4_ solution	Magnesium sulfate led to the participation of gypsum and corrosion of the matrix formed
		Ambient temperature (about 25 °C)	The effect of sodium sulfate was negligible
			Lower w/b ratio improved the pore structure and led to higher resistance to sulfate attack
[65]	Class F-FA (60%)	Duration: 90 days	Bio-additives were used
	GGBFS	5% Na_2_SO_4_ solution	Compressive strength loss was 2.95%
	Na_2_SiO_3_	Renewed every 30 days	Maximum density loss was 3.91% for all specimens with bio-additives compared to 13.97% without bio-additives
	NaOH		Porosity decreased with the addition of bio-additives, before and after immersions in the solution
			Weight loss was 0.68%
[40]	Class F-FA (0–50%)	Duration: 180 days	The weight increased with increasing fly ash replacement in Na_2_SiO_3_ and MgSO_4_ solutions
	GGBFS (50–100%)	5% Na_2_SO_4_ solution	The immersion in sodium and magnesium sulfates showed an increase in weight
	Na_2_SiO_3_	5% MgSO_4_ solution	With the increase of fly ash under sulfate attack, the compressive strength decreased
	NaOH	Renewed every 30 days	The replacement by 40% of fly ash revealed no gypsum or ettringite formation under sulfate environments
		Ambient temperature	
[63]	Class F-FA	Duration: 274 days	The mass change for samples in sodium sulfate and magnesium sulfate solutions increased initially, then decreased
	Granulated lead smelter slag (GLSS) with FA	5% Na_2_SO_4_ solution, and combined 5% Na_2_SO_4_ and 5% MgSO_4_ solutions	The blended fly ash and slag was the least influenced by an increase of 0.8% and 1.8% after immersion in sodium sulfate and magnesium sulfate solutions, respectively
	Na_2_SiO_3_	Renewed every 60 days at room temperature (about 23 °C)	The leaching of sodium hydroxide under the attack of sodium sulfate had a significant effect on the compressive strength
	NaOH	Continuous immersion or wetting-drying and heating-cooling conditions	

[*] FA: fly ash.

## Data Availability

Data are contained within the article.
